# Towards understanding of heat effects in metallic glasses on the basis of macroscopic shear elasticity

**DOI:** 10.1038/srep23026

**Published:** 2016-03-15

**Authors:** Y. P. Mitrofanov, D. P. Wang, A. S. Makarov, W. H. Wang, V. A. Khonik

**Affiliations:** 1 Department of General Physics, State Pedagogical University, Lenin St. 86, Voronezh, 394043 Russia; 2 Institute of Physics, Chinese Academy of Sciences, Beijing 100190, PR China

## Abstract

It is shown that all heat effects taking place upon annealing of a metallic glass within the glassy and supercooled liquid states, i.e. heat release below the glass transition temperature and heat absorption above it, as well as crystallization-induced heat release, are related to the macroscopic shear elasticity. The underlying physical reason can be understood as relaxation in the system of interstitialcy-type ”defects” (elastic dipoles) frozen-in from the melt upon glass production.

The basic feature of all glasses consists in thermal effects occurring upon heating. In general, these effects appear as *i*) heat release below the glass transition temperature *T*_*g*_, *ii*) heat absorption above *T*_*g*_ and *iii*) crystallization-induced heat release^1^,^2^,^3^,^4^,^5^. In the literature, these effects are discussed mostly from the phenomenological viewpoint while their physical nature remains elusive for all types of glasses. It is not even known whether the heat effects occurring within the glassy state and those taking place upon crystallization are determined by the same mechanism or not. In this paper, the origin of heat effects is considered using experimental data taken on a typical Zr-based metallic glass.

A well known interpretation of heat effects in metallic glasses is based on the free volume notion, which states that a change of the “free volume” below or above *T*_*g*_ (within the non-crystalline state) yields release or absorption of the internal energy^6^,^7^. However, this hypothesis provides only qualitative interpretation of calorimetric data^7^,^8^ and was repeatedly criticized (e.g. ref. 9). Recently, rather clear evidence was obtained that the heat release and heat absorption in metallic glasses below the crystallization onset temperature are determined by the macroscopic shear elasticity, which is probed by measurements of the shear modulus^10^,^11^,^12^,^13^. The underlying physical reason can be understood as relaxation in the system of dumbbell interstitial-type “defects”, which can be treated as elastic dipoles. Here we first show for a typical Zr-based glass that the same conclusion applies for the *largest* thermal effect—crystallization-induced heat release and, therefore, *all* thermal phenomena are determined by shear modulus relaxation. Thus, our study outlines a new promising approach for the understanding of thermal effects and nature of “defects” in glasses.

The general idea that the unrelaxed shear modulus *G* (i.e. *G* measured during the time much smaller than the characteristic time of structural relaxation) represents the key physical quantity controlling the relaxation kinetics of supercooled liquids and glasses was introduced long ago^14^ and now becomes more and more accepted^15^,^16^,^17^,^18^. The reason consists in the basic understanding that elementary atomic rearrangements take place during the time of about inverse Debye frequency so that the reaction of the surrounding structure is purely elastic and, therefore, controlled by the unrelaxed shear modulus. The activation energy for these rearrangements then becomes proportional to *G*^14^,^15^,^16^,^17^. Besides that, the unrelaxed shear modulus constitutes a thermodynamic parameter because it is defined as the second derivative of the free energy with respect to the shear strain^19^.

Meanwhile, the instantaneous shear modulus is the key physical quantity in the Interstitialcy theory proposed by Granato^20^,^21^. The theory was inspired by the observation^22^ that dumbbell (split) interstitials (=interstitialcies) introduced into copper by soft neutron irradiation at low temperatures lead to a strong decrease of the shear modulus so that a hypothetical defect concentration of 2–3% should provide a vanishing *G*, which is a signature of liquid^23^. To date, it is generally accepted that dumbbell interstitials exist in all main crystalline structures and represent the basic state of interstitials in metals^24^,^25^,^26^. It was suggested that melting of simple metallic crystals takes place through rapid multiplication of interstitialcy defects^20^,^21^,^27^, which retain their individuality in the liquid state^28^. Subsequent melt quenching freezes the defects in solid glass, which become inherent structural elements rather than “defects” of the structure. Then, structural relaxation of glass can be interpreted in terms of the change of interstitialcy “defect” concentration. This hypothesis leads to rather numerous successful interpretations of various relaxation phenomena in metallic glasses at different conditions (for a review, see ref. 29 and papers cited therein).

In particular, the interstitialcy formation enthalpy *H* is proportional to the shear modulus, *H* = *α*Ω*G*, (Ω is the volume per atom, the invariant of interstitialcy elastic strain field *α*^30^ is assumed to be close to unity^20^,^21^,^27^) and, therefore, any change of the “defect” concentration should lead to heat release or heat absorption depending on the sign of “defect” concentration change. This can be precisely monitored by measurements of the shear modulus because the latter is exponentially dependent on the “defect” concentration *c*, i.e.^20^,^21^


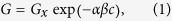


where *G*_*x*_ is the shear modulus of the maternal crystalline state (reference crystal thereafter, i.e. the one, which was used for glass production), *β* is the dimensionless shear susceptibility^20^,^21^ and *α* is the same as in the above expression for the interstitialcy formation enthalpy. The quantity *β* is defined as negative ratio of the 4^*th*^-order shear modulus to the 2^*nd*^-order shear modulus^20^,^21^,^30^ (the latter is commonly referred to as simply “shear modulus”). Experimental investigations on different metallic glasses show^10^,^11^,^12^,^13^,^29^ that *β*-values are about 17–20, depending on chemical composition. Analytically, the heat flow (heat per unit time and unit mass) due to a change of the “defect” concentration within the framework of this approach is given as^10^


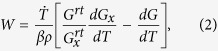


where *G*^*rt*^ and 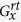 are the shear moduli of glass and reference crystal at room temperature, respectively, 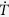 is the heating rate and *ρ* is the density. According to Eq. (1), the ratio 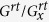 in the relationship (2) defines room-temperature concentration *c*_*rt*_ of “defects” frozen-in upon glass production as 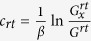. If the melt is quenched to another temperature, this ratio must be taken for this temperature.

It was shown that Eq. (2) gives a good description of the heat release below *T*_*g*_ as well as the heat absorption in the *T*_*g*_-region for several metallic glasses^10^,^11^,^12^,^13^,^29^. However, an issue of major importance is the lack of understanding whether this equation can be also applied to the *most pronounced* thermal effect in metallic glasses—heat release occurring upon crystallization. There is only a general expectation for that and theoretical estimates^30^,^31^,^32^ implying that since the interstitialcy “defect” concentration is decreasing upon crystallization, the enthalpy related them will be also released and, therefore, Eq. (2) may be valid.

The experimental verification of this hypothesis poses a problem, which essentially lies in shear modulus measurements near and above the crystallization onset temperature *T*_*x*_ and, if such measurements are possible, in their quality. Indeed, Eq. (2) shows that calculations of the heat flow require very precise input *G*-data because *W* is determined by the difference between two derivatives of the shear moduli *G* and *G*_*x*_ with respect to temperature. In the experiments mentioned above^10^,^11^,^12^,^13^, the error of precise *G*-measurements rapidly increases at high temperatures due to the exponential growth of the internal friction and related exponential decrease of the shear viscosity that makes automatic measurements at temperatures slightly above *T*_*g*_ eventually impossible. We solved this problem (see below) and performed high precision shear modulus measurements on a typical Zr-based metallic glass not only far above *T*_*g*_ but also in the whole crystallization range. We found that Eq. (2) provides a very good description of all thermal effects—heat release below *T*_*g*_, heat absorption in the *T*_*g*_-region due to structural relaxation and, which is most important, heat release upon crystallization. The results suggest that all heat effects have common structural origin, which can be understood as a change of the concentration of interstitialcy-type “defects” (elastic dipoles).

## Results

**Figure 1:**
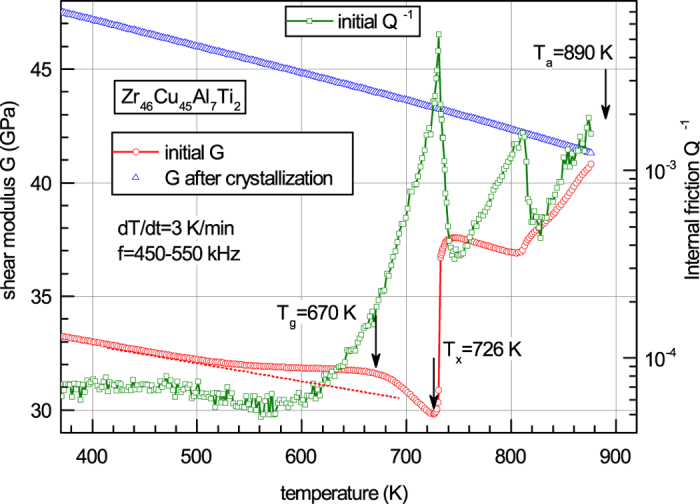
Temperature dependences of the shear modulus and internal friction (green squares) of bulk glassy *Zr*_46_*Cu*_45_*Al*_7_*Ti*_2_. Shear modulus data for the initial state (red circles) and after crystallization (blue triangles) at *T*_*a*_ = 890 K are shown. The calorimetric glass transition temperature *T*_*g*_ and crystallization onset temperature *T*_*x*_ are indicated by the arrows. Structural relaxation-induced shear modulus increase at temperatures *T* > 500 K and shear softening in the range *T*_*g*_ < *T* < *T*_*x*_ as well as strong crystallization-induced increase of *G* at *T* > *T*_*x*_ constitute the basic features of the elastic shear behavior of metallic glasses. The two internal friction peaks reflect the two stages of glass crystallization.

**Figure 2:**
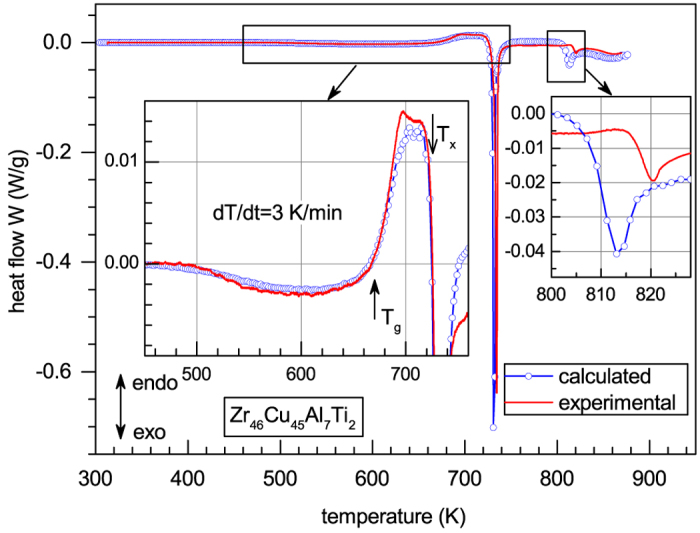
A comparison of experimental heat flow (solid red curve) with the calculations performed using Equation (2). It is seen that Eq. (2) gives a very good reproduction of all basic thermal effects—structural relaxation-induced heat release below *T*_*g*_, heat absorption above *T*_*g*_ and the first crystallization-induced heat release above *T*_*x*_. According to Eq. (2), these heat effects are controlled by relaxation of the shear modulus. The underlying physical reason can be understood as relaxation in the system of internal “defects”, which can be identified as dumbbell interstitials or elastic dipoles.

Figure 1 shows temperature dependences of the shear modulus of this glass in the initial state and after full crystallization at *T*_*a*_ = 890 K together with the corresponding internal friction data taken at a rate of 3 K/min. The shear modulus of the initial sample first linearly decreases with temperature due to the anharmonicity up to ≈500 K while at higher temperatures there is an upward change of *G* (i.e. above the dotted line) due to structural relaxation, which is accompanied by heat release (see Fig. 2 below). At about calorimetric 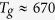 K (shown by the arrow), *G* starts to rapidly decrease, which is common to metallic glasses^10^,^13^,^29^. Crystallization begins at *T*_*x*_ ≈ 726 K leading to a very fast increase of the shear modulus by 24.4%. The internal friction *Q*^−1^ of the initial sample increases very rapidly at *T* > 600 K, reaches the maximum at *T* ≈ *T*_*x*_, abruptly falls down next and runs up to the second maximum after that. The second *Q*^−1^ peak at ≈810 K clearly indicates the phase transition in the crystalline state, which, however, results in minor changes of the shear modulus. It is to be noticed that *Q*^−1^ near *T*_*x*_ is several times smaller than that in other metallic glasses at similar frequencies^13^. It is the relatively low internal friction as well as the high viscosity, which allow performing measurements far above *T*_*g*_ and in the whole crystallization range.

The solid red curve in Fig. 2 gives the experimental heat flow obtained by DSC for the same glass at the same heating rate. A significant structural relaxation-induced heat release corresponding to the increase of the shear modulus is clearly observed in the same range of 500 K < *T* < 660 K (see left inset). The heat flow gradually transforms into heat absorption above *T*_*g*_ and finally leads to a big crystallization heat release peak above *T*_*x*_, which is typical of metallic glasses^4^,^5^.

**Figure 3:**
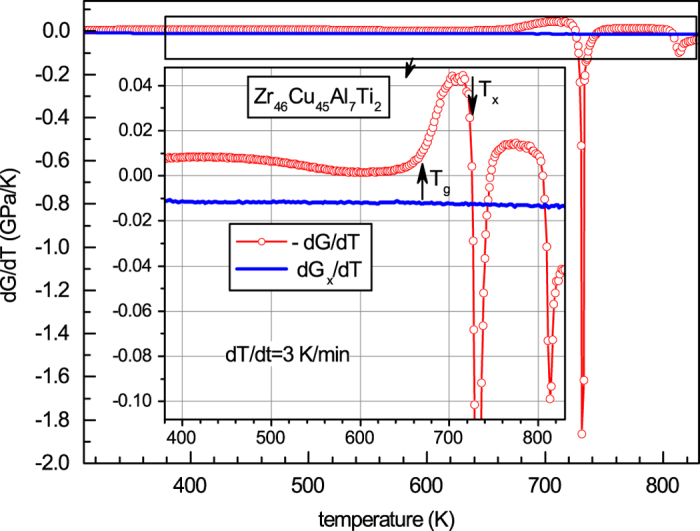
Temperature derivatives of the shear modulus in the initial state (red circles) and after crystallization (solid blue curve). It is seen that while *dG*_*x*_/*dT* is nearly constant, −*dG*/*dT* clearly correlates with the heat flow shown in Fig. 2.

Figure 3 shows temperature dependencies of the temperature derivative of the shear modulus in the initial state taken with the opposite sign (−*dG*/*dT*) and temperature derivative of the shear modulus after crystallization (*dG*_*x*_/*dT*). While *dG*_*x*_/*dT* is almost temperature independent, one can point out a close correlation between −*dG*/*dT* and experimental heat flow curve given in Fig. 2.

Thus, both terms in Eq. (2) are now available and one can use them to calculate the heat flow. This is done in Fig. 2, which gives *W*(*T*)-curve calculated using Eq.(2) with *ρ* = 7021 kg/m^3^, 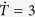 K/min, *G*(*T*), *G*_*x*_(*T*)-dependences shown in Fig. 1 and the shear susceptibility *β* = 18 determined as the least-square fit to the experimental heat flow curve. It is to be noticed that this *β*-value is quite close to the shear susceptibilities determined for other metallic glasses^10^,^11^,^12^,^13^. Figure 2 convincingly demonstrates that Eq. (2) gives an excellent description of the experimental heat release below *T*_*g*_, heat absorption above *T*_*g*_ and crystallization-induced heat release. In particular, the height and temperature of crystallization heat release peak coincide with the experiment within less than 10% and ≈3 K, respectively. The calculation also roughly reproduces the heat release upon 2^*nd*^ stage of crystallization at about 820 K (right inset in Fig. 2), but the agreement with the experiment is worse.

## Discussion

Our experiment conclusively shows that there is a fundamental relationship of the heat flow of a Zr-based metallic glass in the initial state with the shear moduli and their temperature derivatives of this glass in the amorphous and crystalline states. The excellent fit of Eq. (2) to the experimental heat flow (excluding the 2^*nd*^ crystallization peak) provides support that the heat effects due to both structural relaxation and crystallization of glass are intrinsically connected with the relaxation in the system of frozen-in dumbbell interstitial-type “defects”. Heat release below *T*_*g*_ is then conditioned by a decrease of the “defect” concentration (manifested by the increase of the shear modulus)^10^,^29^ while the heat absorption above *T*_*g*_ in the supercooled liquid state can be considered as a result of the increase of the “defect” concentration (revealed as shear softening) towards the metastable equilibrium^29^.

On the other hand, interstitialcy defects represent a particular case of “elastic dipoles”—atomic configurations with the local symmetry, which is lower than that of surrounding matrix^33^. Elastic dipoles interact with external stress and define certain amount of frozen-in elastic energy. Crystallization of glass leads to a decrease of the “defect” concentration and corresponding release of their formation enthalpy. Accepting that dumbbell interstitials are in fact elastic dipoles, one can calculate the kinetics of heat flow due to dissipation of their elastic energy^30^,^34^. This approach (see Eq. (13) in ref. 34) describes structural relaxation- and crystallization-induced heat effects shown in Fig. 2 equally good if compared with Eq. (2). This confirms that dumbbell interstitials can be indeed considered as elastic dipoles and the observed thermal effects are due to the dissipation of their stored elastic energy. The major decrease of the concentration of elastic dipoles takes place during the first crystallization stage explaining the most pronounced exothermal heat reaction. Since a only small part of “defects” remains in the structure after that, rather poor correspondence between calculated and experimental heat flow data for the 2^*nd*^ crystallization stage is quite understandable.

It is worthy of notice that the heat flow given by Eq. (2) can be presented in another useful form. The difference between the enthalpies of glass and reference crystal is given as


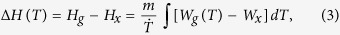


where *W*_*g*_ is the heat flow from glass and the heat flow from crystal *W*_*x*_ can be considered as a constant according to the Dulong-Petit law. At room temperature *T*^* rt*^ and slightly above it, structural relaxation is absent and the heat flow from glass equals to that from crystal so that *W*_*g*_ − *W*_*x*_ = 0. This is actually seen in the inset of Fig. 2 and for the glass under consideration takes place in the range *T *^*rt*^ ≤ *T* < 480 K. The underlying physical reason consists in the equality of the heat capacities of glass and reference crystal in the temperature range with no structural relaxation (e.g. refs 35,36). At higher temperatures, the difference *W*_*g*_ − *W*_*x*_ equals to the heat flow due to a change of the “defect” concentration as given by Eq.(2). Then, Equation (3) after integration from *T*^*rt*^ to a current temperature *T* can be rewritten as


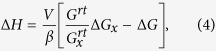


where *V* is the volume and Δ*G*_*x*_, Δ*G* are the changes of the corresponding shear moduli due to heating from *T*^*rt*^ to *T*. Equation (4) explicitly shows that the difference between the enthalpies of glass and reference crystal is conditioned by *i*) room-temperature shear moduli of the initial glass and reference crystal and *ii*) changes of these moduli taking place upon heating. While the change of the shear modulus of crystal is solely of anharmonic nature, the change of the shear modulus of glass includes both anharmonic and relaxation components (this is actually clearly seen in Fig. 1). Since temperature coefficients of the shear moduli of glass and reference crystal in the absence of structural relaxation are equal^37^, the corresponding anharmonic shear modulus changes are also equal. The relaxation contribution into the shear modulus of glass is determined by a change of the concentration of frozen-in interstialcy “defects” (elastic dipoles), which is given by Eq. (1) (see also ref. 29 and papers cited therein).

Overall, the simple relationship (4) clearly indicates the relationship of heat effects in metallic glasses with the macroscopic shear elasticity of glass and reference crystal. However, anharmonic components of the shear moduli of glass and crystal have to be considered only in order to provide a correct calculation of the “defect” concentration *c* upon changing the temperature (since the shear modulus of glass in Eq. (1) is temperature dependent not only because of relaxation change of *c* but also due to temperature change of *G*_*x*_) and the heat effects considered above are conditioned solely by the relaxation change of the “defect” concentration. The fact that Eq. (2) correctly describes all heat effects including those occurring upon crystallization provides new evidence that the difference between the internal energies of glass and reference crystal is determined mainly by the elastic strain fields of interstitialcy “defects” (elastic dipoles), which relax both during structural relaxation and crystallization of glass, as suggested earlier^30^,^31^.

In summary, all three thermal effects in the metallic glass under investigation—structural relaxation-induced heat release below *T*_*g*_, heat absorption above *T*_*g*_ as well as crystallization-induced heat release—are related with the changes of the shear moduli of glass and reference crystal, which take place upon heating. The underlying physical reason can be understood as thermoactivated relaxation in the system of interstitialcy-type “defects”, which behave as elastic dipoles frozen-in upon glass production.

## Methods

Glassy *Zr*_46_*Cu*_45_*Al*_7_*Ti*_2_ (at.%) under investigation was prepared by melt suction and X-ray checked to be entirely amorphous. Differential scanning calorimetry (DSC) was performed using a Perkin Elmer DSC 8000 instrument in flowing Ar atmosphere. Measurements of the heat flow were done for initial glassy and crystallized states. To remove the uncertainties related with the baseline of the DSC instrument, the heat flow of crystallized sample was subtracted from the heat flow of initial glassy sample.

Shear modulus measurements were carried out by the electromagnetic acoustic transformation (EMAT) method, which is based on the Lorentz interaction of surface currents in a metallic sample induced by the exciting coil with the external magnetic field^10^. The basic advantage of this method consists in the absence of direct acoustic contact between the sample and exciting/signal coils. The transverse resonant vibration frequency *f* (about 450–550 kHz depending on heat treatment) of sample (5 × 5 × 2 mm^3^) was determined as the frequency corresponding to the maximum of the shear vibration amplitude upon frequency scanning. The latter was automatically performed every 10–30 s depending on temperature. The shear modulus was calculated as 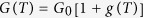, where *G*_0_ = 33.61 GPa^38^ is the shear modulus at room temperature and 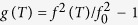 is the change of the shear modulus with *f*_0_ being the resonant frequency at room temperature. The relative error in the calculation of absolute shear modulus is estimated to be ≈5 × 10^−3^ while the relative error for the shear modulus *change* is unprecedented—from ≈5 ppm at temperatures far below *T*_*g*_ to about 100 ppm at high temperatures far above *T*_*g*_. The internal friction was calculated as 
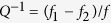
, where *f*_1_ and *f*_2_ were determined upon scanning as the frequencies corresponding to the vibration amplitude equal to 0.707 × *A*_*r*_ with *A*_*r*_ being the resonant vibration amplitude^33^. In order to perform precise measurements at high internal friction (i.e. deep in the supercooled liquid state far above *T*_*g*_ as well as in the crystallization range), we carried out an extensive modification of the setup. The measurements were carried out in vacuum of about 0.013 Pa.

The main problem consisted in the choice of glass for the experiment. We tested quite a few Pd- and Zr-based metallic glasses and found that most of them are unsuitable for precise automatic *G*-measurements because of the high internal friction and low shear viscosity (the latter sometimes leads to a change of sample’s dimensions even under the action of its own weight) above *T*_*g*_, which eventually result in the loss of signal tracking. The situation becomes slightly better upon using low heating rates, which lead to a decrease of the internal friction and increase of the shear viscosity. However, low heating rates strongly promote oxidation of small samples (tens of milligrams) during DSC measurements even at increased Ar flow rate and while applying other efforts for preventing oxidation (oxidation of bulk samples during shear modulus measurements is not significant). Samples’ oxidation heavily corrupts DSC thermograms complicating their comparison with calculated heat flow from *G*-data using Eq. (2). Eventually, after a number of trials-and-errors, we ended up with *Zr*_46_*Cu*_45_*Al*_7_*Ti*_2_ glass, which combines low *Q*^−1^ and high viscosity above *T*_*g*_ together with relatively low oxidation propensity.

## Additional Information

**How to cite this article**: Mitrofanov, Y. P. *et al*. Towards understanding of heat effects in metallic glasses on the basis of macroscopic shear elasticity. *Sci. Rep.* **6**, 23026; doi: 10.1038/srep23026 (2016).
